# Reduced Red Blood Cell Deformability in Vivax Malaria

**DOI:** 10.1093/infdis/jiae490

**Published:** 2024-10-07

**Authors:** Jeyamalar T Thurai Rathnam, Matthew J Grigg, Arjen M Dondorp, Timothy William, Megha Rajasekhar, Giri Rajahram, Julie A Simpson, Bridget E Barber, Nicholas M Anstey

**Affiliations:** Centre for Epidemiology and Biostatistics, Melbourne School of Population and Global Health, University of Melbourne, Melbourne, Victoria, Australia; Global and Tropical Health Division, Menzies School of Health Research and Charles Darwin University, Darwin, Northern Territory, Australia; Infectious Diseases Society Sabah–Menzies School of Health Research Clinical Research Unit, Queen Elizabeth Hospital, Kota Kinabalu, Sabah, Malaysia; Mahidol Oxford Tropical Medicine Research Unit, Faculty of Tropical Medicine, Mahidol University, Bangkok, Thailand; Centre for Tropical Medicine and Global Health, Nuffield Department of Medicine, University of Oxford, Oxford, United Kingdom; Infectious Diseases Society Sabah–Menzies School of Health Research Clinical Research Unit, Queen Elizabeth Hospital, Kota Kinabalu, Sabah, Malaysia; Subang Jaya Medical Centre, Subang Jaya, Malaysia; Centre for Epidemiology and Biostatistics, Melbourne School of Population and Global Health, University of Melbourne, Melbourne, Victoria, Australia; Global and Tropical Health Division, Menzies School of Health Research and Charles Darwin University, Darwin, Northern Territory, Australia; Infectious Diseases Society Sabah–Menzies School of Health Research Clinical Research Unit, Queen Elizabeth Hospital, Kota Kinabalu, Sabah, Malaysia; Department of Medicine, Queen Elizabeth Hospital II Sabah, Ministry of Health, Kota Kinabalu, Sabah, Malaysia; Centre for Epidemiology and Biostatistics, Melbourne School of Population and Global Health, University of Melbourne, Melbourne, Victoria, Australia; Centre for Tropical Medicine and Global Health, Nuffield Department of Medicine, University of Oxford, Oxford, United Kingdom; Global and Tropical Health Division, Menzies School of Health Research and Charles Darwin University, Darwin, Northern Territory, Australia; Infectious Diseases Society Sabah–Menzies School of Health Research Clinical Research Unit, Queen Elizabeth Hospital, Kota Kinabalu, Sabah, Malaysia; Infection and Inflammation Program, QIMR Berghofer Medical Research Institute, Brisbane, Queensland, Australia; Global and Tropical Health Division, Menzies School of Health Research and Charles Darwin University, Darwin, Northern Territory, Australia; Infectious Diseases Society Sabah–Menzies School of Health Research Clinical Research Unit, Queen Elizabeth Hospital, Kota Kinabalu, Sabah, Malaysia

**Keywords:** *Plasmodium vivax*, red blood cell deformability, pathogenesis, spleen, *Plasmodium falciparum*

## Abstract

Reduced deformability of both infected and uninfected red blood cells (RBCs) contributes to pathogenesis in *Plasmodium falciparum* malaria. Whole-blood RBC deformability (RBC-D) is not well characterized in *Plasmodium vivax* malaria. We used a laser-assisted optical rotational cell analyzer to measure the RBC-D in fresh whole-blood samples from Malaysian patients with vivax malaria (n = 25). Deformability of whole-blood RBCs, the vast majority of which were uninfected, was reduced in vivax malaria compared with controls (n = 15), though not to the same degree as in falciparum malaria (n = 90). Reduced RBC-D may contribute to the pathogenesis of vivax malaria, including splenic retention of uninfected RBCs.

## Teaser

Deformability of whole-blood red blood cells, the vast majority of which were uninfected, was reduced in vivax malaria compared with controls, though not as much as in falciparum malaria. Reduced red blood cell deformability may contribute to vivax malaria pathogenesis.

*Plasmodium vivax* usually causes uncomplicated malaria but can cause severe disease [1]. The pathogenesis of *P. vivax*, including its rheopathobiology, is not fully understood [2, 3]. In *Plasmodium falciparum* and *Plasmodium knowlesi* malaria, the deformability of both infected and uninfected red blood cells (RBCs) is reduced, each contributing to reduced RBC flow through the microvasculature and to impaired organ perfusion [4, 5]. Reduced deformability of RBCs in malaria from both of these species has also been associated with anemia [5, 6], thought likely to be through enhanced biomechanical retention of infected and uninfected RBCs in the interendothelial slits in the splenic red pulp [6, 7].

In vivax malaria, the rheopathobiology is more complex than in malaria from other *Plasmodium* species. Studies of single RBCs infected with late-ring and trophozoite stages of *P. vivax* demonstrated increased deformability [8, 9], although RBCs infected with late-stage schizonts become spherical, reducing their ability to flow through narrow capillary beds [10]. Furthermore, the collective deformability of large numbers of RBCs present in clinical whole-blood samples, the vast majority of which are uninfected in vivax malaria [2], is not well characterized [3, 11].

Adequately powered studies assessing RBC deformability (RBC-D) using the laser-assisted optical rotational cell analyzer have not been undertaken in vivax malaria. To determine whether RBC-D is reduced in vivax malaria, we used this analyzer to measure the RBC-D in fresh whole-blood samples from patients with vivax malaria and compared results with those in healthy controls and patients with falciparum malaria.

## METHODS

### Study Sites, Patients, and Study Procedures

Patients hospitalized with malaria were enrolled as part of a prospective observational study at Queen Elizabeth Hospital, a tertiary referral hospital in Kota Kinabalu, Malaysia [5, 12]. The Malaysian Ministry of Health mandates hospitalization for malaria from all *Plasmodium* species. Patients were enrolled if they were aged >12 years, within 18 hours of commencing antimalarial treatment, nonpregnant, and had no major comorbid conditions or concurrent illnesses. Severe malaria was diagnosed according to modified World Health Organization criteria [1, 12]. Healthy controls were visitors or relatives of patients with malaria admitted to Queen Elizabeth Hospital, with no history of fever in the past 48 hours, and with a blood film negative for malaria parasites.

Venous blood was collected in ethylenediaminetetraacetic acid for automated blood counts, mean corpuscular volume (MCV), parasite quantitation on Giemsa-stained blood slide, polymerase chain reaction confirmation of *Plasmodium* species, and measurement of RBC-D. RBC-D was measured on enrollment with a laser-assisted optical rotational cell analyzer (Lorrca MaxSis; Mechatronics) and expressed as an elongation index (EI), as described elsewhere [4, 5]. With this method, whole blood was added to a highly viscous medium (5% polyvinylpyrrolidine in phosphate-buffered saline), and the RBC suspension was sheared between 2 concentric rotating cylinders at a constant temperature of 37°C [5, 6]. RBC-D was assessed at shear stresses (SSs) of 1.7 and 30 Pa; the former are encountered in the capillaries [4]. Altered EIs at SSs of 30 Pa reflect changes in cell geometry, including the ratio of surface area to volume, and approximate values encountered by RBCs passing through interendothelial slits in the splenic red pulp [4, 5].

### Statistical Analysis

Statistical analysis was performed using R software (version 4.1.2). The distribution of EI was compared between patients with *P. vivax* malaria and both the controls and patients with *P. falciparum* malaria, using the Mann-Whitney *U* test. Associations were assessed between EI at SSs of 1.7 and 30 Pa and parasite count, the percentage of schizonts, lactate, platelets, MCV, admission hemoglobin, plasma cell-free hemoglobin, or hemoglobin nadir, using Spearman correlation coefficients. Linear regression was used to compare EIs between patients with *P. vivax* malaria and controls, after adjusting for MCV.

### Ethics Statement

The studies were approved by the ethics committees of the Malaysian Ministry of Health and the Menzies School of Health Research, Darwin, Australia. Informed written consent was provided by all participating adults and by the parent or guardian of any participant aged <18 years.

## RESULTS

### Patients

A total of 25 patients with *P. vivax* malaria were enrolled and compared with 90 patients with falciparum malaria and 15 controls. RBC-D in patients with falciparum malaria and controls has been previously reported [5]. Baseline demographic, clinical, and hematological characteristics are shown in Table 1. Overall, 100 patients (77%) were male, and the median age was 27 years (interquartile range [IQR], 19–41 years; range, 13–62 years). Of the patients with malaria, 2 (8%) with *P. vivax* and 8 (9%) with *P. falciparum* met modified World Health Organization research criteria for severe malaria [12].

**Table 1.** Epidemiological and Clinical Characteristics and Red Blood Cell Deformability of Patients With Malaria and Controls at Queen Elizabeth Hospital

Characteristic	Median Value (IQR)^a^		
	Controls (n = 15)	Patients With *P. vivax* Malaria (n = 25)	Patients With *P. falciparum* Malaria (n = 90)
Age, y	38 (22–45)	21 (18–41)	27 (19–39)
Age range, y	19–58	13–61	13–62
Male sex, no. (%)	11 (73)	20 (80)	69 (77)
Parasite count, parasites/µL	…	5439 (2928– 10 462)	10 211 (2932–33 265)
Parasite stage, %			
Rings	…	27.4 (0.0–63.8)^b^	100 (100–100)^c^
Trophozoite	…	72.6 (33.5–99.7)^b^	0.0 (0.0–0.0)^c^
Schizont	…	0.0 (0.0–0.4)^b^	0.0 (0.0–0.0)^c^
Hemoglobin, g/dL	14.4 (13.3–15.4)^d^	12.6 (11.4–14.3)	13.3 (11.3–14.4)
MCV, fL	86.3 (79.5–89.1)^d^	82.3 (75.8–85.7)	80.4 (75.6–85.7)
MCV <80 fL, no. (%)	4 (33)	44 (49)	10 (40)
Nadir hemoglobin, g/dL	…	11.4 (10.4–12.3)	11.6 (10.3–12.8)
Lactate, µmol/L	…	1.320 (0.900–1.605)^e^	1.210 (0.940–1.570)^f^
RBC-D at SS of 1.7 Pa	0.203 (0.178–0.222)	0.196 (0.160–0.214)	0.182 (0.163–0.198)
RBC-D at SS of 30 Pa	0.583 (0.576–0.590)	0.543 (0.518–0.572)	0.518 (0.481–0.557)
Duration of preceding fever, d	…	5 (4–7)	5 (3–7)
Time from start of treatment, h	…	4.4 (0.0–9.1)	5.8 (0.0–12.4)

Abbreviations: IQR, interquartile range; MCV, mean corpuscular volume; NA, not assessed; *P. falciparum, Plasmodium falciparum*; *P. vivax, Plasmodium vivax*; RBC-D, red blood cell deformability; SS, shear stress.

^a^Data represent median (IQR) unless otherwise specified.

^b^Data missing for 1 patient.

^c^Data missing for 4 patients.

^d^Data missing for 3 controls.

^e^Data missing for 2 patients.

^f^Data missing for 7 patients.

### RBC-D Findings

**Figure 1.**
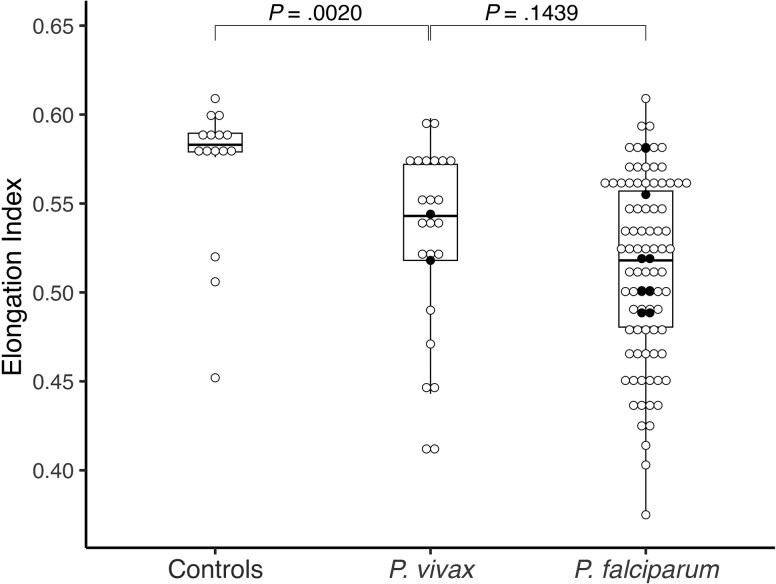
Red blood cell (RBC) deformability in patients with vivax and falciparum malaria. Deformability profile of whole peripheral blood RBCs from patients with vivax malaria (*Plasmodium vivax*; n = 23 nonsevere and n = 2 severe), falciparum malaria (*Plasmodium falciparum*; n = 82 nonsevere and n = 8 severe), and healthy controls (n = 15), as measured with a laser-assisted optical rotational cell analyzer at a shear stress of 30 Pa. A decrease in elongation index reflects a decrease in overall RBC deformability. Open circles represent nonsevere malaria; filled circles, severe malaria [1]; boxes, interquartile range; and horizontal black lines, median.

At an SS of 30 Pa, the median RBC-D was reduced in patients with vivax malaria compared with controls (median EI [IQR] for patients with vivax malaria vs controls, 0.543 [0.518–0.572] vs 0.583 [0.576–0.590]; *P* = .002) (Table 1 and Figure 1). This difference in RBC-D between patients with vivax malaria and controls remained significant after controlling for MCV (*P* = .02; Supplementary Table 1). The reduction in RBC-D in vivax malaria was not as great as in falciparum malaria (Figure 1) [5]; however, the difference in RBC-D between patients with vivax and falciparum malaria was moderate (median EI [IQR] for patients with falciparum malaria, 0.518 [0.481–0.557]; *P* = .14). In patients with vivax malaria, there was no correlation between RBC-D at an SS of 30 Pa and parasite count, the percentage of schizonts, lactate, admission hemoglobin, plasma cell-free hemoglobin, hemoglobin nadir, or MCV (Supplementary Tables 2 and 3).

At a lower SS of 1.7 Pa, there was no difference in median RBC-D between patients with vivax malaria and controls (*P* = .38; Table 1). In patients with vivax malaria, RBC-D at 1.7 Pa was positively correlated with MCV (Spearman ρ = 0.5260; *P* = .007), as also seen in patients with falciparum malaria (Supplementary Table 3). There was no correlation with parasite count, the percentage of schizonts, plasma cell-free hemoglobin, or the hemoglobin nadir (Supplementary Table 2).

## DISCUSSION

Whole-blood RBC-D is reduced in acute vivax malaria, though not to the same degree as seen in falciparum malaria. Because >99.5% of RBCs in peripheral blood were uninfected in these patients, our findings indicate that it is the deformability of uninfected RBCs that is reduced in vivax malaria.

In contrast to falciparum and knowlesi malaria, where both uninfected and infected RBCs have reduced deformability [5, 6], infection with *P. vivax* results in divergent effects on the deformability of infected and uninfected RBCs. RBCs infected with late-ring [9] and trophozoite stages [8, 9] of *P. vivax* have increased deformability, related to an increase in the ratio of surface area to volume in infected RBCs [8, 9]. However, RBCs infected with early-ring and schizont stages have reduced deformability [9]. Our study shows that uninfected RBCs also have reduced deformability in vivax malaria. The mechanisms underlying the reduced deformability of uninfected RBCs in vivax malaria are not known but may be related to heme-induced oxidative damage of the RBC membrane [13], reduced nitric oxide in vivax malaria [14], host anti-RBC antibodies [15], or as-yet-unidentified host and parasite products found in plasma [2, 6].

In vivax malaria there is a paucity of cytoadherence of infected RBCs within the microvasculature [2], the major mechanism of reduced microvascular perfusion found in falciparum malaria. We speculate that impaired deformability of uninfected RBCs may contribute to reduced capillary blood flow in vivax malaria, including in higher-stringency organ capillary beds such as the lung, though likely not to the same extent as in falciparum malaria. At a high SS (30 Pa), approximating values encountered by RBCs passing through interendothelial slits in the splenic red pulp [4, 5], the reduced RBC-D is likely to contribute to the retention of uninfected RBCs in the spleen. Indeed, congestion of uninfected RBCs in the splenic red pulp has been recently identified as the primary cause of splenomegaly in vivax malaria [7].

Our findings also show that the effect of RBC volume (MCV) on RBC-D is apparent at a low SS (1.7 Pa) in both vivax and falciparum malaria but not at a higher SS. Hemoglobinopathies, such as thalassemia, also reduce RBC-D. Hemoglobin phenotyping was not done in the current study; however, it is unlikely that hemoglobinopathies were an important confounder because the difference remained significant after controlling for MCV. We were unable to determine any relationship between reduced RBC-D and anemia, as previously found in falciparum [5, 6] and knowlesi [5] malaria, but we may have been underpowered for this analysis.

In conclusion, whole-blood RBC-D is reduced in vivax malaria. This may contribute to splenic retention of uninfected RBCs in *P. vivax* infection and to vivax pathogenesis.

## Supplementary Data

Supplementary materials are available at *The Journal of Infectious Diseases* online (http://jid.oxfordjournals.org/). Supplementary materials consist of data provided by the author that are published to benefit the reader. The posted materials are not copyedited. The contents of all supplementary data are the sole responsibility of the authors. Questions or messages regarding errors should be addressed to the author.

## Supplementary Material

jiae490_Supplementary_Data

## Notes

***Acknowledgments.*** We thank the Sabah malaria research team nursing and laboratory staff; the hospital director and clinical staff at Queen Elizabeth Hospital; and the director general of health, Malaysia, for permission to publish the deformability studies.

***Financial support.*** This work was supported by the National Health and Medical Research Council of Australia (grant 496600; project grant 1045156; investigator grants 2017436 to M. J. G., 2016792 to B. E. B., and 1196068 to J. A. S.; and fellowship 1135820 to N. M. A.) and the Wellcome Trust of Great Britain (A. M. D.).
