# Supplementary material for: Reduced Red Blood Cell Deformability in Vivax Malaria
Source: J Infect Dis. 2024 Oct 7;231(3):e566–9. doi: 10.1093/infdis/jiae490 (PMC11911793; doi:10.1093/infdis/jiae490)
Supplement: jiae490_Supplementary_Data [file jiae490_supplementary_data.docx]

**Supplementary material**

Supplementary Tables 1 and 3 include data analysed for patients with vivax and falciparum malaria, and also knowlesi malaria from the same study cohort. The RBC-deformability (elongation index) data and MCV (mean corpuscular volume) comparison between patients with non-severe and severe knowlesi malaria were previously reported [5]. The correlations between RBC-deformability and MCV **(Suppl. Table 3)** for knowlesi malaria were not previously reported.

**Supplementary Table 1:** Linear regression analyses to explore the association between RBC-deformability (elongation index (EI) at shear stress 1.7 Pa and 30 Pa) and healthy individuals (controls) or malaria patients (*P. falciparum*, *P. knowlesi* or *P. vivax*), adjusted for MCV.

| RBC-deformability | Model covariates | *P. falciparum* (n=90) | |  | *P. knowlesi* (n=82) | |  | *P. vivax* (n=25) | |
| --- | --- | --- | --- | --- | --- | --- | --- | --- | --- |
|  |  | Estimate  (95% CI) | p-value |  | Estimate  (95% CI) | p-value |  | Estimate  (95% CI) | p-value |
| EI at 1.7 Pa | MCV (fL) | 0.0011  (0.0004, 0.0018) | 0.001 |  | 0.0024  (0.0017, 0.0031) | <0.001 |  | 0.0025  (0.0012, 0.0037) | <0.001 |
|  | Malaria (vs controls) | -0.0098  (-0.0295, 0.0098) | 0.322 |  | 0.0033  (-0.0143, 0.0209) | 0.713 |  | 0.0049  (-0.0181, 0.0279) | 0.667 |
| EI at 30 Pa | MCV (fL) | 0.0003  (-0.0008, 0.0014) | 0.578 |  | 0.0013  (0.00004, 0.0026) | 0.044 |  | -0.0002  (-0.0023, 0.0019) | 0.822 |
|  | Malaria (vs controls) | -0.0560  (-0.0871, -0.0249) | 0.001 |  | -0.0478  (-0.0823, -0.0134) | 0.007 |  | -0.0442  (-0.0825, -0.0060) | 0.025 |

Controls (reference group): n=12 (3 with missing MCV data); CI, confidence interval.

**Supplementary Table 2:** Spearman’s correlation analyses to determine the relationship between pathological parameters and RBC-deformability (elongation index (EI) at shear stresses of 1.7 Pa and 30 Pa respectively), for patients infected with *P. vivax* malaria (n=25).

| Pathological parameters | EI at 1.7 Pa | |  | EI at 30 Pa | |
| --- | --- | --- | --- | --- | --- |
|  | Correlation coefficient | p-value |  | Correlation coefficient | p-value |
| Admission haemoglobin (g/dL) | 0.4125 | 0.040 |  | 0.1477 | 0.481 |
| Nadir haemoglobin (g/dL) | 0.3326 | 0.104 |  | 0.1692 | 0.419 |
| Plasma cell-free haemoglobin (g/dL) | 0.0235 | 0.911 |  | 0.1681 | 0.422 |
| Parasite count (/µL of blood) | 0.3563 | 0.080 |  | 0.0427 | 0.839 |
| Percentage schizonts (%)^a^ | 0.0644 | 0.765 |  | 0.0866 | 0.687 |
| Lactate (µmol/L)^b^ | 0.6435 | 0.001 |  | 0.2358 | 0.279 |
| Platelets (x1000/µL of blood) | -0.3447 | 0.092 |  | 0.1882 | 0.368 |

^a^n=24 (1 with missing percentage schizont data), ^b^n=23 (2 with missing lactate data)

**Supplementary Table 3:** Spearman’s correlation between RBC-deformability (elongation index (EI) at shear stress levels 1.7 Pa and 30 Pa) and MCV for patients with falciparum, knowlesi, and vivax malaria.

| Correlation with MCV (fL) | *P. falciparum* (n=90) | |  | *P. knowlesi* (n=82) | |  | *P. vivax* (n=25) | |
| --- | --- | --- | --- | --- | --- | --- | --- | --- |
|  | Correlation coefficient | p-value |  | Correlation coefficient | p-value |  | Correlation coefficient | p-value |
| EI at 1.7 Pa | 0.2876 | 0.006 |  | 0.6137 | <0.001 |  | 0.5260 | 0.007 |
| EI at 30 Pa | 0.1122 | 0.292 |  | 0.2246 | 0.042 |  | -0.0385 | 0.855 |
